# Mixed storm in SARS‐CoV‐2 infection: A narrative review and new term in the Covid‐19 era

**DOI:** 10.1002/iid3.838

**Published:** 2023-04-26

**Authors:** Basil Mohammed Alomair, Hayder M. Al‐Kuraishy, Ali I. Al‐Gareeb, Ali K. Al‐Buhadily, Athanasios Alexiou, Marios Papadakis, Majed Ayed Alshammari, Hebatallah M. Saad, Gaber El‐Saber Batiha

**Affiliations:** ^1^ Department of Medicine, College of Medicine, Internal Medicine and Endocrinology Jouf University Al‐Jouf Saudi Arabia; ^2^ Department of Clinical Pharmacology and Medicine, College of Medicine Al‐Mustansiriya University Baghdad Iraq; ^3^ Department of Clinical Pharmacology, Medicine, and Therapeutic, Medical Faculty, College of Medicine Al‐Mustansiriyah University Baghdad Iraq; ^4^ Department of Science and Engineering Novel Global Community Educational Foundation Hebersham New South Wales Australia; ^5^ AFNP Med Wien Austria; ^6^ Department of Surgery II, University Hospital Witten‐Herdecke University of Witten‐Herdecke Wuppertal Germany; ^7^ Department of Medicine Prince Mohammed Bin Abdulaziz Medical City Sakaka Al‐Jouf Saudi Arabia; ^8^ Department of Pathology, Faculty of Veterinary Medicine Matrouh University Marsa Matruh Egypt; ^9^ Department of Pharmacology and Therapeutics, Faculty of Veterinary Medicine Damanhour University Damanhour Egypt

**Keywords:** Covid‐19, cytokine storm, inflammatory storm, lipid storm, mixed storm, oxidative storm

## Abstract

Coronavirus disease 2019 (Covid‐19) is caused by a novel severe acute respiratory syndrome coronavirus virus type 2 (SARS‐CoV‐2) leading to the global pandemic worldwide. Systemic complications in Covid‐19 are mainly related to the direct SARS‐CoV‐2 cytopathic effects, associated hyperinflammation, hypercytokinemia, and the development of cytokine storm (CS). As well, Covid‐19 complications are developed due to the propagation of oxidative and thrombotic events which may progress to a severe state called oxidative storm and thrombotic storm (TS), respectively. In addition, inflammatory and lipid storms are also developed in Covid‐19 due to the activation of inflammatory cells and the release of bioactive lipids correspondingly. Therefore, the present narrative review aimed to elucidate the interrelated relationship between different storm types in Covid‐19 and the development of the mixed storm (MS). In conclusion, SARS‐CoV‐2 infection induces various storm types including CS, inflammatory storm, lipid storm, TS and oxidative storm. These storms are not developing alone since there is a close relationship between them. Therefore, the MS seems to be more appropriate to be related to severe Covid‐19 than CS, since it develops in Covid‐19 due to the intricate interface between reactive oxygen species, proinflammatory cytokines, complement activation, coagulation disorders, and activated inflammatory signaling pathway.

## Graphical abstract

SARS‐CoV‐2 infection induces various storm types including cytokine storm (CS), inflammatory storm, lipid storm, thrombotic storm (TS), and oxidative storm. These storms are not developing alone since there is a close relationship between them. Therefore, the mixed storm seems to be more appropriate to be related to severe Covid‐19 than CS, since it develops in Covid‐19 due to the intricate interface between reactive oxygen species, proinflammatory cytokines, complement activation, coagulation disorders, and activated inflammatory signaling pathway

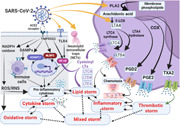

## 1 INTRODUCTION

The Coronavirus disease 2019 (Covid‐19) is an existing pandemic disease caused by a novel severe acute respiratory syndrome coronavirus virus type 2 (SARS‐CoV‐2).^1^, ^2^ SARS‐CoV‐2 is a single strand RNA virus from *Betacoronavireadae* family and has a genetic relationship with other coronaviruses like bat coronavirus, SARS‐CoV and Middle East Respiratory Syndrome coronavirus virus.^3^ SARS‐CoV‐2 primarily emerged in Wuhan, China, leading to unidentified viral pneumonia termed Wuhan pneumonia. Later, this virus was renamed as a novel coronavirus virus 2019. Afterward, for a short period, the World Health Organization warned of this disease as a pandemic and changed the name of this virus to SARS‐CoV‐2.^4^ Covid‐19 is a primary respiratory disease leading to respiratory symptoms indistinguishable from other flues like illness presented with fever, headache, dry cough, dyspnea, myalgia, joint pain, and anosmia.^2^, ^5^ Additional studies and researches showed that Covid‐19 may cause systemic complications, including acute kidney injury, thromboembolic disorders, and gastrointestinal and neurological complications.^6^, ^7^, ^8^ Generally, Covid‐19 is frequently asymptomatic in around 85% of affected patients. Though, 15% of the affected patients presented with severe dyspnea and serious respiratory symptoms due to the development of acute lung injury (ALI). Furthermore, 5% of Covid‐19 patients prerequisite hospitalization and intensive care unit (ICU) admission due to the progression of acute respiratory distress syndrome (ARDS).^9^ Severely affected Covid‐19 patients may necessitate invasive oxygen supplementation and mechanical ventilation.^9^, ^10^

Management of Covid‐19 patients is largely supportive and symptomatic relief since specific anti‐SARS‐CoV‐2 was not yet developed despite the development of effective vaccines.^11^ Notably, many repurposed agents like remdesivir and favipiravir were encompassed in diverse therapeutic protocols in the management of Covid‐19.^11^, ^12^ These agents did not fashion actual therapeutic eradication of SARS‐CoV‐2, and enduring for novel anti‐SARS‐CoV‐2 agents is a type of challenge nowadays.^11^

Most patients with mild and moderate Covid‐19 are resolved and responded to the usual symptomatic treatments.^13^ However, severe Covid‐19 may advance with the development of systemic complications. The underlying cause of systemic complications is mainly related to the direct SARS‐CoV‐2 cytopathic effects, associated hyperinflammation, hypercytokinemia and the development of cytokine storm (CS).^10^, ^14^ Many recent studies indicated that Covid‐19 complications are developed due to the propagation of oxidative and thrombotic events which may progress to a severe state called oxidative storm and thrombotic storm (TS), respectively.^15^ Furthermore, defect in the lymphocytic cytolytic activity triggers prolonged activation of macrophages resulting in the development of macrophage activation syndrome (MAS).^16^ Notably, MAS is characterized by immune hyper‐activation and high levels of proinflammatory cytokines and is regarded as one of the most important cause of mortality in Covid‐19.^16^

In normal immune response against SARS‐CoV‐2 infection, monocytes, macrophages, neutrophils, and dendritic cells express pattern recognition receptor (PRR) which recognizes pathogen‐associated molecular patterns (PAMPs).^4^ One of the most important PRRs is a toll‐like receptor (TLR) which chiefly recognized extracellular PAMPs and to a lesser extent intracellular PAMPs.^4^ Activated TLRs provoke nuclear factor kappa B (NF‐κB) which stimulates interferon (INF) release and subsequent antiviral response.^17^ However, the intracellular damage‐associated molecular patterns (DAMPs) are recognized by a set of cytosolic sensors like nod‐like receptor pyrin (NLRP) which increase transcription of a cytoplasmic multiprotein complex called inflammasomes to form an immune sensor called NLRP3 inflammasome.^17^ Activated NLRP3 inflammasome converts pro‐caspase‐1 to caspase‐1 which converts prointerleukin1β (pro‐IL1β) to IL1β which is an important proinflammatory cytokine involved in the progression of CS.^16^ Normally, virally‐infected cells are identified and destroyed by CD8+ cells of adaptive immunity and natural killer (NK) cells of innate immunity through the perforin‐mediated process with induction of apoptosis.^18^ Besides, to avoid unnecessary immune activation, the antigen‐presenting cells and cytotoxic T cells (CD8+) undergo apoptosis. This normal immune response is developing in patients with asymptomatic or mild Covid‐19.^19^, ^20^, ^21^ If there is a defect in the lymphocyte functions, the CD8 and NK cells are unable to destroy virally infected cells with subsequent prolonged immune activation and higher interaction between adaptive and innate immune responses.^22^ In this state, exaggerated immune response and release of proinflammatory cytokines like tumor necrosis factor‐alpha (TNF‐α), IL‐1β and IL‐6 are developed causing CS leading to severe and critical Covid‐19.^16^, ^22^

The risk factors for the development of CS and exaggerated immune response in Covid‐19 patients are the old age group, male sex and associated comorbidities like hypertension, diabetes, and obesity.^23^, ^24^, ^25^, ^26^ Chronic preexisting metabolic conditions during viral infection increase disease severity and mortality.^27^, ^28^, ^29^ The altered metabolic environment and impaired immune system reinforced by hypertension, obesity, and diabetes may exacerbate the severity of the disease among infected patients.^23^ Though, the current understanding of the connection between hypertension, obesity, and diabetes and related cardiovascular and renal complications with Covid‐19 is developing.^30^, ^31^, ^32^ Many studies have highlighted the role of angiotensin‐converting enzyme 2 (ACE2) receptor expressions and systemic inflammation in promoting SARS‐CoV‐2 infection and disease severity.^33^, ^34^ Even though several pharmacological interventions for these chronic metabolic disorders also seem to affect ACE2 expression, it is not clear whether they have any role in the disease severity.^7^, ^23^

Therefore, components and mediators of innate and adaptive immune responses contribute mutually to the regulation of immune response during Covid‐19 (Table 1).

**Table 1:** Components and mediators of innate and adaptive immune responses.

Immune responses	Immune cells and mediators
Innate immune response	Monocytes, macrophages, neutrophils, dendritic cells and interferon (INF).
Adaptive immune response	NLR Family Pyrin Domain Containing 3 (NLRP3) inflammasome, tumor necrosis factor‐α, interleukin (IL)‐1β and IL‐6

Therefore, the present narrative review aimed to elucidate the interrelated relationship between different storm types in Covid‐19. Different published articles with findings from prospective, retrospective, and clinical studies were selected to verify the development of various storm types in Covid‐19.

## 2 CS IN COVID‐19

CS is a vivid picture that refers to the flaring up of immunoinflammatory response with sudden exaggerated release of proinflammatory cytokines.^35^, ^36^ Ferrara et al.^37^ in 1993 was the first one who used the CS term as a complication of graft‐versus‐host disease. Later on, this term was more frequently used by many studies in relation to various viral infections.^38^, ^39^ After that, the CS term started appearing more commonly in different scientific literatures.

At the beginning of the Covid‐19 pandemic, Mehta et al.^40^ first described CS in the pathogenesis of SARS‐CoV‐2 infection. In the Covid‐19 era, the CS term captured the attention of the scientific community and public in scientific literatures and social media. Notably, hypercytokinemia which is a different entity from that of CS refers to the increasing levels of proinflammatory cytokines in different infectious and inflammatory disorders.^2^, ^40^ Sudden uncontrolled release of proinflammatory cytokines and chemokines is the most suitable definition for CS.^35^, ^39^, ^41^

The potential mechanism of CS in SARS‐CoV‐2 infection is complex and related to intricate interaction between SARS‐CoV‐2 and immune cells.^8^, ^42^ During SARS‐CoV‐2 infection and associated cytopathic injury, PAMPs are released which activate PRRs. The interaction between PAMPs and PRRs triggers innate immune response for expression and release of proinflammatory cytokines, adhesion molecules, chemokines, and inflammatory signaling pathways like NF‐κB.^43^, ^44^, ^45^

Entry of SARS‐CoV‐2 into the host cell is mediated through ACE2 which is highly expressed in different cell types including lung alveolar cells.^2^, ^42^ ACE2 is involved in the metabolism and conversion of angiotensin II (AngII) to Ang1‐7.^46^ Downregulation of ACE2 by SARS‐CoV‐2 leads to an increasing level of proinflammatory AngII and reduction of anti‐inflammatory Ang1‐7.^47^, ^48^ It has been shown that augmentation of AngII in SARS‐CoV‐2 infection can activate several inflammatory signaling pathways like NF‐κB, NLRP3 inflammasome and a disintegrin and metalloprotease 17 (ADAM17) with subsequent release of proinflammatory cytokines.^49^, ^50^ SARS‐CoV‐2 can directly activate NF‐κB and NLRP3 inflammasome with the succeeding release of proinflammatory cytokines like IL‐6 and TNF‐α.^51^, ^52^ Furthermore, the binding of IL‐6 to its IL‐6 receptor trigger other inflammatory signaling pathways like signal transducer and activator of transcription 3 (STAT3) which is involved in the release of proinflammatory cytokines.^17^, ^53^ Both STAT3 and NF‐κB can induce the release of chemokines, monocyte chemoattractant protein 1, IL‐6 and IL‐8.^17^, ^54^, ^55^

SARS‐CoV‐2 stimulates the Th1 immune response with the release of IL‐6 and granulocyte/macrophage colony‐stimulating factor (GM‐CSF) which activates CD14/CD16 to release IL‐6 and other proinflammatory cytokines.^14^, ^56^ Activation of TLR4 by SARS‐CoV‐2 can induce exaggeration of immune response and release of proinflammatory cytokines with weak INF‐γ response.^30^ In addition, activated neutrophils during SARS‐CoV‐2 infection may form the neutrophil extracellular traps (NETs) with further release of proinflammatory cytokines.^30^ Taken together, impairment of attained immune response and exaggerated innate immune response in Covid‐19 triggers uncontrolled immune activation and release of a huge amount of proinflammatory cytokines causing CS.^14^, ^30^, ^34^

It has been shown that CS development is linked with Covid‐19 severity, fatality and poor clinical outcomes.^57^ Remy et al.^58^ found that Covid‐19 severity was associated with the suppression of both adaptive and innate immune responses. A systematic review and meta‐analysis observed that CS and lymphopenia were linked with Covid‐19 severity.^59^ Analysis of the cytokine profile in 41 Covid‐19 with CS showed that most of the proinflammatory cytokines were increased and correlated with high mortality.^60^ Similarly, a retrospective study that included 150 Covid‐19 patients with CS illustrated that IL‐6 was the most predictor cytokine in relation to Covid‐19 severity and mortality.^61^ The binding of SARS‐CoV‐2 to the ACE2 induces the release of DAMPs which induce immune cells to release proinflammatory cytokines. A huge amount of proinflammatory cytokines trigger the development of CS which causes disseminated intravascular coagulopathy and multiorgan failure and ARDS.^60^, ^61^

Of interest, some inflammatory biomarkers like IL‐6, D‐dimer, and lactate dehydrogenase (LDH) are associated with the development of CS and Covid‐19 severity.^62^ A high IL‐6 serum level is regarded as the main predictor for the progression of CS.^63^ However, exaggerated serum levels of D‐dimer and LDH reflect thrombotic events and tissue injury, respectively.^62^ Dong et al.^64^ proposed a scoring system for SARS‐CoV‐2 infection severity, in which Covid‐19 index = 3 × D‐dimer (µg/L) + 2 × lg ESR (mm/h) − 4 × lymphocytes (×10^9^/L) + 8. Thus, erythrocyte sedimentation rate (ESR), D‐dimer, and lymphocyte count are regarded as the main predictors of Covid‐19 severity.

**Figure 1:**
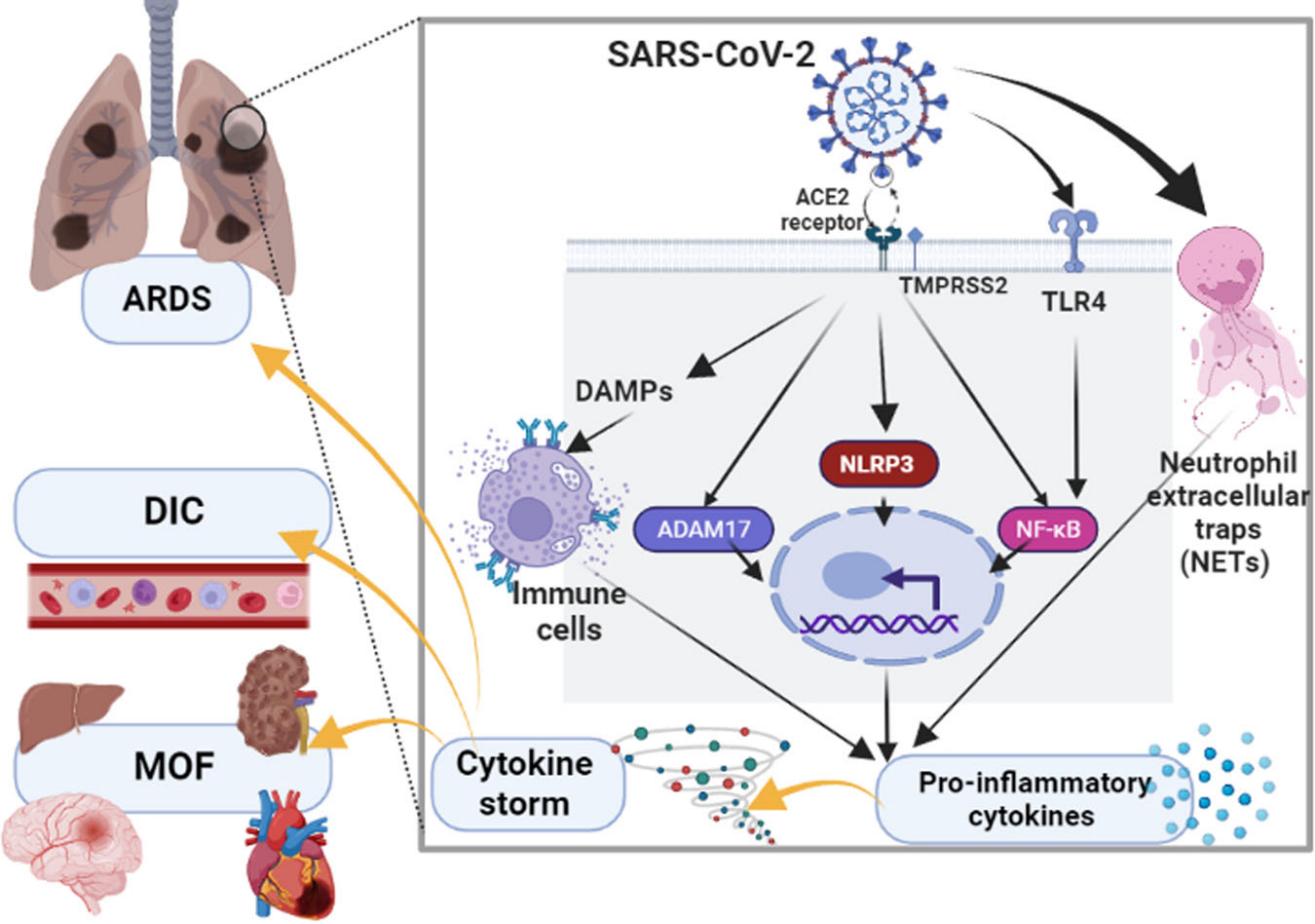
Cytokine storm in Covid‐19: Binding of SARS‐CoV‐2 to the angiotensin‐converting enzyme 2 (ACE2) in association with transmembrane protein protease serine 2 (TMPPSS2) induces the release of damage‐associated molecular patterns (DAMPs) which induce immune cells to release proinflammatory cytokines and activates several inflammatory signaling pathways like nuclear factor kappa B (NF‐κB), nod‐like receptor pyrin 3 (NLRP3) inflammasome and a disintegrin and metalloprotease 17 (ADAM17) with subsequent release of proinflammatory cytokines. SARS‐CoV‐2 activates neutrophils to form the neutrophil extracellular traps (NETs) with further release of proinflammatory cytokines. A huge amount of proinflammatory cytokines trigger the development of cytokine storm which causes disseminated intravascular coagulopathy (DIC) and multiorgan failure (MOF) and acute respiratory distress syndrome (ARDS). Covid‐19, coronavirus disease 2019; SARS‐CoV‐2, severe acute respiratory syndrome coronavirus virus type 2.

These findings confirmed that the development of CS is associated with Covid‐19 severity and mortality due to the development of ALI/ARDS and multiorgan injury (MOI) (Figure 1).

## 3 LIPID STORM IN COVID‐19

### 3.1 Prostaglandin (PG) metabolites

In Covid‐19, activated bioactive lipids like eicosanoids enhance neutrophil activation and recruitment with the development of inflammatory reactions.^55^, ^65^ Of note, both cyclooxygenase (COX) and phospholipase A2 (PLA2) are activated by SARS‐CoV‐2 leading to an increase in the generation of prostaglandin (PG) metabolites.^66^ Targeting of inflammatory lipids may reduce lung inflammation in Covid‐19 patients.^55^ A comparative study comprised 33 Covid‐19 patients and 25 healthy controls illustrated that the fatty acid and bioactive lipid content in the bronchoalveolar lavage (BAL) were higher in Covid‐19 patients compared to the controls.^65^ In addition, COX and lipooxygenase (LOX) contents like PGs and leukotrienes (LTs) were increased significantly in the BAL of Covid‐19 patients compared to the healthy controls. Besides, the antiinflammatory lipids such as resolvin and lipoxin A4 are also increased in the BAL of Covid‐19 patients.^65^, ^67^, ^68^ These findings suggest that proinflammatory and antiinflammatory lipid mediators are highly disturbed in Covid‐19, reflecting the development of lipid storm.

In Covid‐19, high proinflammatory cytokines mainly IL‐1β induce activation of lung COX with subsequent production and release of thromboxane A2 (TXA2) causing alveolar inflammation and bronchoconstriction.^67^ As well, TXA2 promotes platelet activation and increases the risk of pulmonary microthrombosis and the development of ARDS in severely affected Covid‐19 patients.^68^ A prospective study comprising 30 Covid‐19 showed that TXA2 serum level was increased in severely affected Covid‐19 patients and correlated with lung radiological lesions compared to 30 healthy controls.^68^

Similarly, PGD2 induces airway inflammation by activating the recruitment of inflammatory cells including basophils, eosinophils, mastocytes, and lymphocytes. Though, PGE2 leads to the induction of antiinflammatory effects through the inhibition of the release of proinflammatory cytokines from activated macrophages and neutrophils.^66^ In contrast, PGE2 has antiinflammatory effects on the airways.^66^ Therefore, inhibition of COX by nonsteroidal antiinflammatory drugs (NSAIDs) like aspirin and steroids like dexamethasone may limit the progression of lung inflammation and the development of ARDS in Covid‐19 patients.^69^ Of interest, the use of aspirin in severely affected Covid‐19 patients reduces hospital stay and the need for mechanical ventilation through suppression of inflammatory and thrombotic events.^69^ A systematic review according to the evidence from clinical trials illustrated that NSAIDs could be effective against the development of ALI/ARDS in Covid‐19 patients.^70^ In this state, inhibition of prothrombotic TXA2 and proinflammatory PGD2 by dual inhibitors like ramatroban could be effective in reducing pulmonary inflammatory and thrombotic reactions in critically affected Covid‐19 patients.^66^, ^71^ As well, ramatroban is also effective in the prevention of silicosis through the inhibition effects of PG metabolites.^72^ Archambault et al.^73^ found that ramatroban was useful in the reduction of lipid mediators in the BAL of intubated patients with severe Covid‐19.

Moreover, oxylipins are oxygenated natural products formed on demand from polyunsaturated free fatty acids (PUFA) by the action of COX and 5‐Lipoxygenase (LO‐5).^74^ Oxylipins act in paracrine and autocrine fashions; they modulate adipocyte function through the activation of peroxisome proliferators activated receptors (PPARs).^74^, ^75^ Most of the oxylipins are derived from PUFA like linoleic and α‐linoleic acids, though oxylipins are present in higher concentrations in blood and tissues than PUFA.^76^ Oxylipins have anti‐inflammatory and/or proinflammatory effects in different diseases including fatty liver disease, atherosclerosis and Alzheimer's disease.^77^, ^78^, ^79^ Remarkably, oxylipins derived from omega‐6 fatty acids are more proinflammatory than those derived from omega‐3 fatty acids and are involved in vasoconstriction and proliferation.^80^, ^81^ Omega‐3 fatty acids derived from oxylipins have antiinflammatory and vasodilatory effects. During inflammation higher expression of COX and LO‐5 trigger the production and release of oxylipins.^80^, ^82^ In Covid‐19, oxylipins are increased in severely affected patients due to hyperinflammation and oxidative stress.^80^, ^83^ Oxylipin levels are exaggerated in hospitalized Covid‐19 patients in the ICU and positively correlated with markers of macrophage activation. Therefore, proinflammatory oxylipins are augmented in Covid‐19 patients due to defects in the metabolic pathway for the production of antiinflammatory and proresolving oxylipins.^80^, ^83^ As well, glycerophospholipids are glycerol bases phospholipids that are regarded as the main component of the biological membrane.^84^ In severe Covid‐19 there are significant abnormalities in the metabolism of glycerophospholipids. In prospective studies, glycerophospholipids like malonic acid, 1‐methyluronic acid and 3‐hydroxybutyric acid were increased while 1,3‐hydroxybutyric acid was downregulated in severely affected Covid‐19 patients.^85^, ^86^ Thus, dysregulation of glycerophospholipids could be intricate in the pathogenesis of ALI and ARDS in Covid‐19.

**Figure 2:**
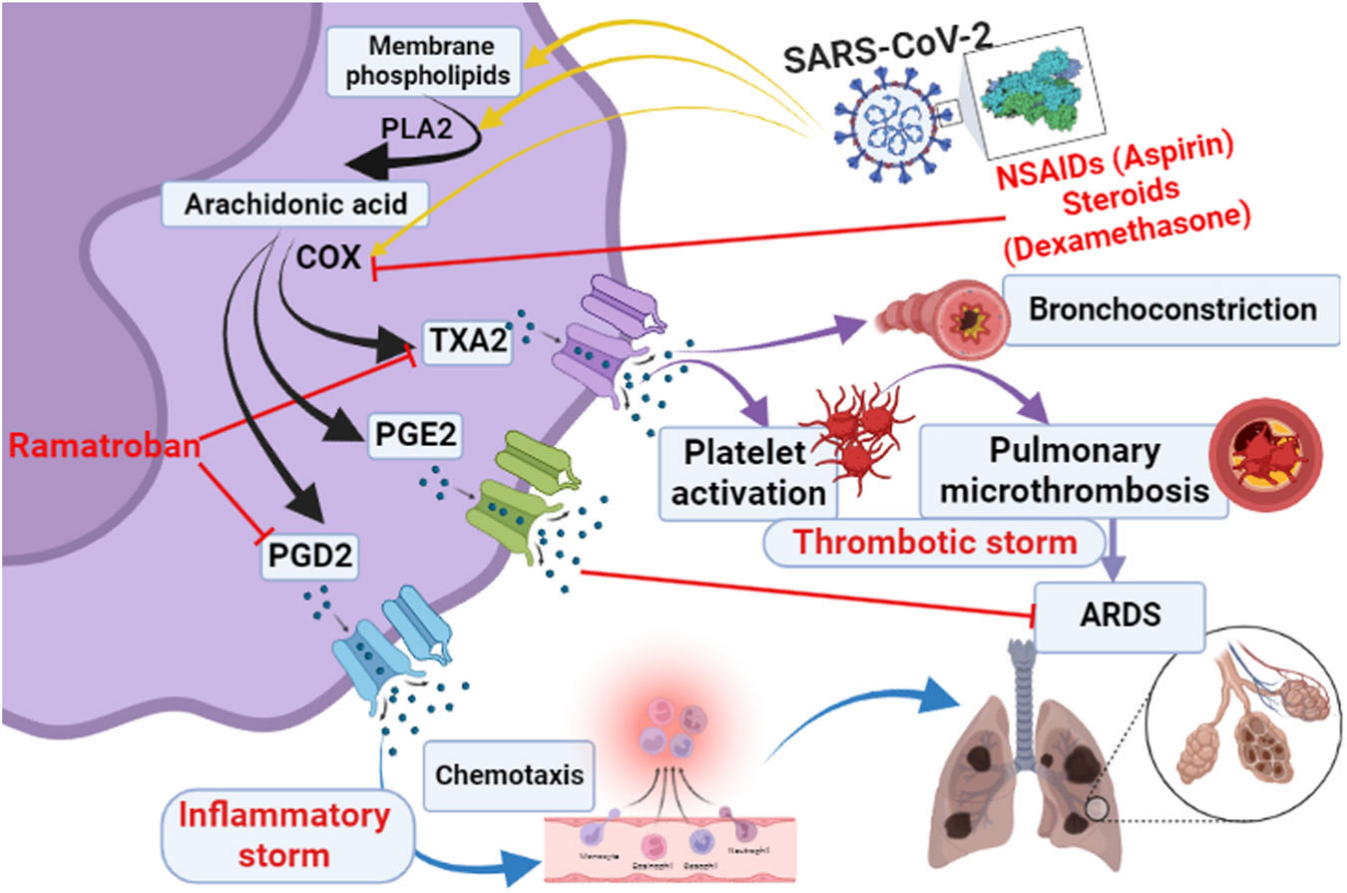
Prostaglandin metabolites in Covid‐19: Phospholipase A2 (PLA2) activates the conversion of membrane phospholipid to arachidonic acid (AA) which is converted by cyclooxygenase (COX) to thromboxane A2 (TXA2), prostaglandin E2 (PGE2) and prostaglandin D2 (PGD2). TXA2 induces thrombosis and PGD2 activates the immune cells while PGE2 has an anti‐inflammatory effect and reduces the development of acute respiratory distress syndrome (ARDS). Prostaglandin D2 (PGD2) induces airway inflammation by activating the recruitment of inflammatory cells. SARS‐CoV‐2 activates phospholipid, AA, and COX.

These findings proposed that PG metabolites are involved in lung and airway inflammation and linked with Covid‐19 severity. Targeting of PG metabolites may ameliorate lung pathology in Covid‐19 (Figure 2).

### 3.2 LT metabolites

LT metabolites notably LTB4 and LTE4 are increased in SARS‐CoV‐2 infection and participate in the induction of immunoinflammatory reactions and propagation of pulmonary dysfunction in Covid‐19 patients.^3^, ^87^ LTs are important eicosanoids produced by many cells including leukocytes. The main class of LTs is the cysteinyl LTs (CysLTs) which are involved in the pathogenesis of allergic rhinitis and asthma.^88^ CysLTs activate specific receptors namely CysLT1R, CysLT2R, purinergic receptor (P2Y12), 2‐oxoglutarate known as a GPR99, and GPR17.^89^, ^90^ These G protein receptors are expressed on many cell types including inflammatory and immune cells.^89^ CysLT1R is highly expressed in the leukocytes and lymphoid cells; CysLT2R is highly expressed in the lung, brain, and leukocytes, P2Y12 is highly expressed in the platelets and airway, GPR17 is mainly expressed by the stem and progenitor cells, and GPR99 is mostly expressed by endothelial and airway mucinous cells.^55^, ^90^, ^91^ In respiratory viral infections, LTB4 is increased to enhance the antiviral response though it causes inflammation and airway injury.^92^ LTB4 is regarded as a chemoattractant for recruitment of the neutrophils and lymphocytes through G‐protein coupled receptors. LTB4 increases the influx of lymphocytes to the lung and airway causing severe lymphopenia, a hallmark of SARS‐CoV‐2 infection pathogenesis.^3^ Besides, LTB4 promotes the activation of macrophages and other inflammatory cells with the release of proinflammatory cytokines and the development of CS in Covid‐19.^3^ High LTs levels in Covid‐19 are associated with poor clinical outcomes and high mortality.^93^ Vorobjeva^93^ and colleagues showed that leukotriene A4 (LTA4) hydrolase which increases the synthesis of LTA4 was increased while the endogenous inhibitor Spink6 was reduced in severely affected Covid‐19 patients. Of interest, SARS‐CoV‐2 can activate LTA4 hydrolase leading to an increase of LTA4 in the lung and airway. Thus, LTA4 hydrolase inhibitors like hydroxamic acid may decrease the risk of pulmonary inflammation in Covid‐19.^94^, ^95^ LTA4 also induces chemoattraction for monocytes and neutrophils.^94^

Moreover, 5LOX which is chiefly expressed in myeloid cells such as macrophages, eosinophils, neutrophils, and mast cells, is also expressed in the fibroblast and lung epithelial cells.^96^, ^97^ The expression of 5LOX is augmented during acute inflammation, for example, TLR4 induces the synthesis of LTs in the activated macrophages via increasing expression of 5LOX.^98^ In turn, LTs provoke the expression of proinflammatory cytokines in the activated macrophages in murine models.^99^ In Covid‐19, complement activation, coagulation disorders, activated inflammatory signaling pathways and proinflammatory cytokines increase the expression of 5LOX with suppression of antiinflammatory resolvin and lipoxins.^100^ Therefore, 5LOX inhibitors like zileuton and PF‐4191834 may decrease the risk of ALI and hyperinflammation in Covid‐19.^100^

**Figure 3:**
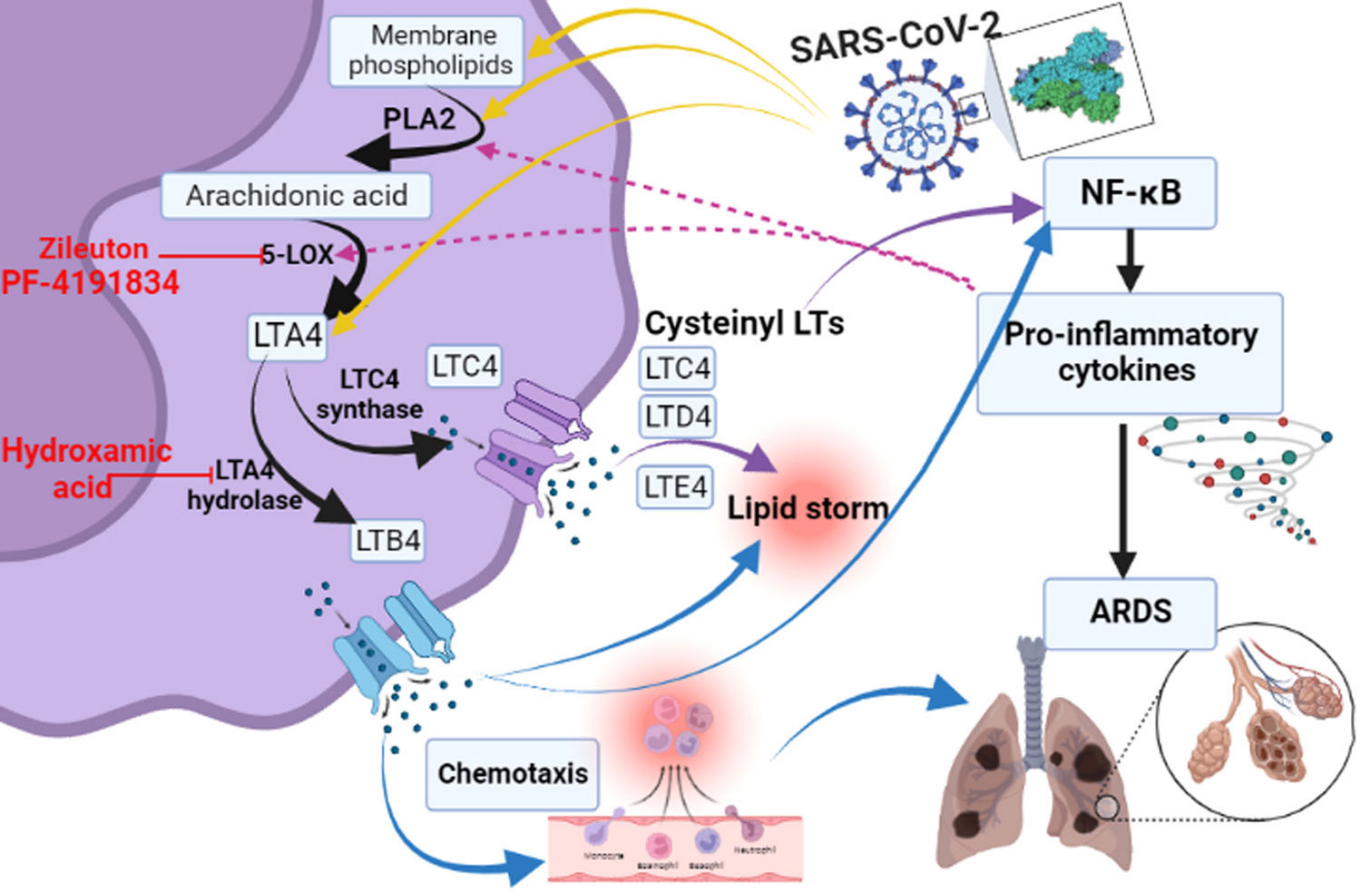
Leukotriene metabolites in Covid‐19: Phospholipase A2 (PLA2) activates the conversion of membrane phospholipid to arachidonic acid (AA) which is converted by 5‐lipooxygenase (5‐LOX) to leukotriene A4 (LTA4) which converted to LTB4 and cysteinyl LTs leading to activation of nuclear factor kappa B (NF‐κB) and development of lipid storm and release of proinflammatory cytokines. In turn, proinflammatory cytokines induce the development of acute respiratory distress syndrome (ARDS). As well, proinflammatory cytokines stimulate the expression of PLA2 and 5‐LOX. Zileuton and PF‐4191834 are 5‐LO inhibitors while hydroxamic acid is an inhibitor of LTA4 hydrolase. Covid‐19, coronavirus disease 2019; LT, leukotriene.

Taken together, PLA2 activates the conversion of membrane phospholipid to AA which is converted by 5‐LOX to LTA4 which is converted to LTB4 and cysteinyl LTs leading to activation of NF‐κB and development of lipid storm and release of proinflammatory cytokines. In turn, proinflammatory cytokines induce the development of ARDS. As well, proinflammatory cytokines stimulate the expression of PLA2 and 5‐LOX,^96^, ^99^ (Figure 3). Targeting of 5LOX and COX by specific inhibitors may attenuate the development of lipid storm in Covid‐19.

### 3.3 Alterations of lipid profile in Covid‐19

It has been shown that changes in lipid profile are linked with Covid‐19 severity. The most often reported changes are a decline in serum cholesterol and apolipoprotein A (ApoA1) levels and increased triglyceride levels. The hyperinflammatory state mediated by the CS perturbs numerous fundamental lipid biosynthesis pathways.^101^ SARS‐CoV‐2 replication is a process that considerably changes the host cell's lipid metabolism program and overuses cell lipid resources during severe infection. Lower high‐density lipoprotein (HDL‐C) and ApoA1 levels are connected with higher severity and mortality rates and with superior levels of inflammatory markers.^101^

Of note, lipids play diverse metabolic roles as structural components, energy resources and signaling mediators in different viral infections.^102^ Numerous immune‐modulatory macrophage regulation pathways depend on lipids and the role of lipid metabolism in pulmonary infections and inflammatory states have been observed.^102^ The effect of different coronaviruses types on lipid metabolism and serum lipid profile items had been observed before the current pandemic. Several studies have shown important changes in the lipid profile of patients with Covid‐19 infection.^102^, ^103^ Furthermore, alterations in lipid profile have been reported ever since the early stages of the Covid‐19 pandemic; most particularly as decreases in cholesterol levels. A retrospective study found decreased levels of total cholesterol (TC) and low‐density lipoprotein (LDL‐c) in 248 Covid‐19 patients.^104^ However, the Covid‐19 patients had lower TC and LDL‐C levels upon admission that would gradually recover as the patients' clinical condition improved.^104^ Besides, retrospective studies showed that low levels of TC and LDL‐C could predict more severe involvement and increased risk of cardiovascular diseases due to changes.^105^, ^106^, ^107^ A systematic review and meta‐analysis showed that lipid profile is associated with both the severity and mortality in Covid‐19 patients. Therefore, the lipid profile may be used for assessing the severity and prognosis of Covid‐19.^108^

It has been believed that infection with SARS‐CoV‐2 decreases Apo‐A1 and HDL‐C levels, both of which relate to the severity of the disease. As well, serum lipidome in Covid‐19 patients highly resembled the membranes of a certain type of membrane‐bound extracellular vesicles, rich in GM‐3.^109^ In a series of 46 Covid‐19 patients, a reduction in 100 lipids was observed, mainly notably in the apolipoproteins related to macrophage regulatory processes.^109^ ApoA, sphingolipids, glycerophospholipids, some steroid precursors, ApoM, and choline were downregulated, the two latter of which were seen in severe involvement. Choline, a precursor of phosphocholine, is a molecule essential in the de novo biosynthesis of phosphatidylcholine the main phospholipid in cell membranes. Thus, a decrease in choline levels might be due to increased macrophage activation and activity.^109^

The underlying mechanisms for the alteration of the lipid profile are related to the modification of both lipid signaling and metabolism to benefit their replication as lipids constitute not only the main structure of membranes but also play important roles as intercellular signaling agents and energy sources for SARS‐CoV‐2.^104^ Replication of SARS‐CoV‐2‐ which enter the cells via endocytosis and use intracellular organelles to produce their different parts requires lipid resources.^104^ Therefore, studying how SARS‐COV‐2 infection affects lipid metabolism and profile might shed light on the correlation between lipid profile and inflammatory processes during Covid‐19.

Notoriously, lipid metabolism can be altered directly by the CS and indirectly by the effect of endothelial dysfunction and associated inflammatory disorders in Covid‐19 patients.^110^ Notable, lipid metabolism is highly disturbed in chronic inflammatory conditions.^111^ Direct SARS‐CoV‐2 interaction with HDL‐C promotes the development of dysfunction HDL‐C which has proinflammatory and proatherogenic effects.^112^ These changes increase the risk for the development of CS by inducing the release of proinflammatory cytokines. In addition, hyperinflammation and the development of a CS as a result of an excessive immune response against SARS‐CoV‐2 may inhibit the expression of peroxisome proliferator activator receptors alpha (PPARα) which involve in the regulation of lipoprotein lipase and peripheral lipid metabolism.^75^ Therefore, PPARα agonist fenofibrate could be effective in the mitigation of inflammatory and oxidative stress disorders.^75^ Studies suggest that omega‐3 derivatives may amend hyper‐inflammation and CS resulting from pulmonary involvement and systemic inflammation.^101^, ^113^ A scoping review illustrated that severe Covid‐19 patients have low levels of omega‐3 in their blood. Omega 3 was estimated to reduce the risk of being positive for SARS‐CoV‐infection and the duration of symptoms, overcome systemic inflammation, and increase the survival rate in Covid‐19 patients.^114^ Omega 3 fatty acid supplementations may have a potential effect in preventing and treating Covid‐19.

These verdicts indicated that lipid profiles are highly dysregulated in Covid‐19 patients leading to the propagation of systemic inflammation and development of systemic inflammation. Targeting this disorder may attenuate hyperinflammation and Covid‐19 associated complications.

## 4 OXIDATIVE STORM IN COVID‐19

To our knowledge, the oxidative storm term was first used by Broxton et al.^115^ to describe how invading pathogens evade immune‐mediated oxidative stress. Later on, in 2020 Kwiatkowska et al.^116^ mentioned that tryptophan metabolites during renal failure can induce oxidative storm with induction of endothelial injury and development of atherosclerosis. Oxidative stress in relation to viral infection was first described by Peterhans in 1979.^117^ It has been shown that respiratory viral infections are associated with the propagation of oxidative stress.^118^ For example, oxidative stress is developed in infection with the influenza virus, respiratory syncytial virus (RSV), and rhinovirus due to the generation of reactive oxygen species (ROS) and expression of nitric oxide synthase 2.^119^ Oxidative stress in viral infections induces further complications through impairment of apoptosis, immune response, and exaggeration of inflammatory response with organ dysfunction.^120^ Induction of ROS during viral infection could be beneficial to some degree in the eradication of phagocytosed viruses by immune cells.^120^ As well, ROS improves signal transduction among different immune cells.^121^ For example, lung alveolar macrophages relatively produce a small amount of ROS for intracellular signaling during respiratory viral infection.^97^, ^121^ Thus, adequate redox homeostasis during viral infection is essential to prevent the development of oxidative stress and storm.

In Covid‐19, oxidative stress is developed due to the overproduction of ROS and reduction of endogenous antioxidant capacity.^15^, ^49^ The risk factors for the development of oxidative stress in Covid‐19 are old age, male sex, black race, obesity, and diabetes mellitus.^30^, ^122^, ^123^ Exaggerated oxidative stress leads to the propagation of oxidative storm which increases Covid‐19 severity.^75^, ^124^ A case‐control study that included 24 patients with severe Covid‐19 and 24 healthy control subjects showed that serum levels of oxidative stress markers were higher in Covid‐19 patients compared to the healthy controls.^124^ Total oxidant status and malondialdehyde (MDA) were increased in Covid‐19 patients compared to the healthy controls.^124^, ^125^ Of note, activated macrophages in response to SARS‐CoV‐2 infection produce ROS, and together with reactive nitrogen species (RNS) can induce endothelial dysfunction and lung epithelial cell injury.^126^ As well, nicotinamide adenine dinucleotide phosphate (NADPH) oxidase is also stimulated during SARS‐CoV‐2 infection‐causing generation ROS/RNS with the propagation of oxidative burst.^126^ It has been shown that viral infections including SARS‐CoV‐2 infection can suppress the endogenous antioxidant system including glutathione transferase, superoxide dismutase, and catalase in human alveolar cells causing epithelial injury.^127^, ^128^

Higher oxidative stress in Covid‐19 also reduces serum albumin which acts as an antioxidant and antiinflammatory. In a retrospective study, low albumin serum level was correlated with Covid‐19 severity.^129^ Indeed, the development of oxidative stress is related to the dysregulation of the renin‐angiotensin system (RAS).^130^, ^131^ Notably, AngII induces activation of NADPH oxidase with the generation of ROS.^132^, ^133^ Therefore, a higher AngII level in Covid‐19 could be the proposed cause for the development of oxidative stress in Covid‐19.^15^ Activation of NADPH oxidase in Covid‐19 leads to activation of the immune system, impairment of the antioxidant system, induction of thrombosis, and RAS imbalance.^126^, ^134^

Moreover, a recent advance in vitamin D research points out that this vitamin has valuable effects on numerous body systems. Both 25 dihydroxy vitamin D (25(OH)2D) and its active hormonal form, 1,25‐dihydroxy vitamin D (1,25(OH)2D) are essential for human physiological functions, including damping down inflammation and the excessive intracellular oxidative stresses.^135^ Vitamin D is one of the important controllers of systemic inflammation, oxidative stress, and mitochondrial respiratory function. The molecular and cellular actions of 1, 25(OH)2D decelerate oxidative stress, and cell and tissue damage.^136^ Hypovitaminosis D enhances oxidative stress and systemic inflammation. The interaction of 1,25(OH)2D with its intracellular receptors modulates vitamin D‐dependent gene transcription and activation of vitamin D‐responsive elements, which activates multiple second messenger systems.^137^ Novel understandings of vitamin D‐related advances in metabolomics, transcriptomics, and epigenetics, in relation to its ability to control oxidative stress in conjunction with micronutrients, vitamins, and antioxidants, following normalization of serum 25(OH)D and tissue 1,25(OH)2D concentrations, likely to promise cost‐effective better clinical outcomes in humans.^136^, ^137^ Remarkably, vitamin D supplementation stimulated the binding of the SARS‐CoV‐2 cell entry receptor ACE2 to angiotensin II receptor type 1, reducing the number of virus particles that could attach to ACE2 and enter the cell.^138^ Furthermore, vitamin D deficiency seems to be associated with increased COVID‐19 risk. In contrast, vitamin D can normalize mitochondrial dynamics, which would improve oxidative stress, proinflammatory state, and cytokine production. Also, vitamin D reduces RAS activation and, subsequently, reduces ROS generation and improves the prognosis of SARS‐CoV‐2 infection.^139^ Therefore, vitamin D is regarded as an important factor that could improve disease severity through its anti‐inflammatory and antioxidant effects.^139^

**Figure 4:**
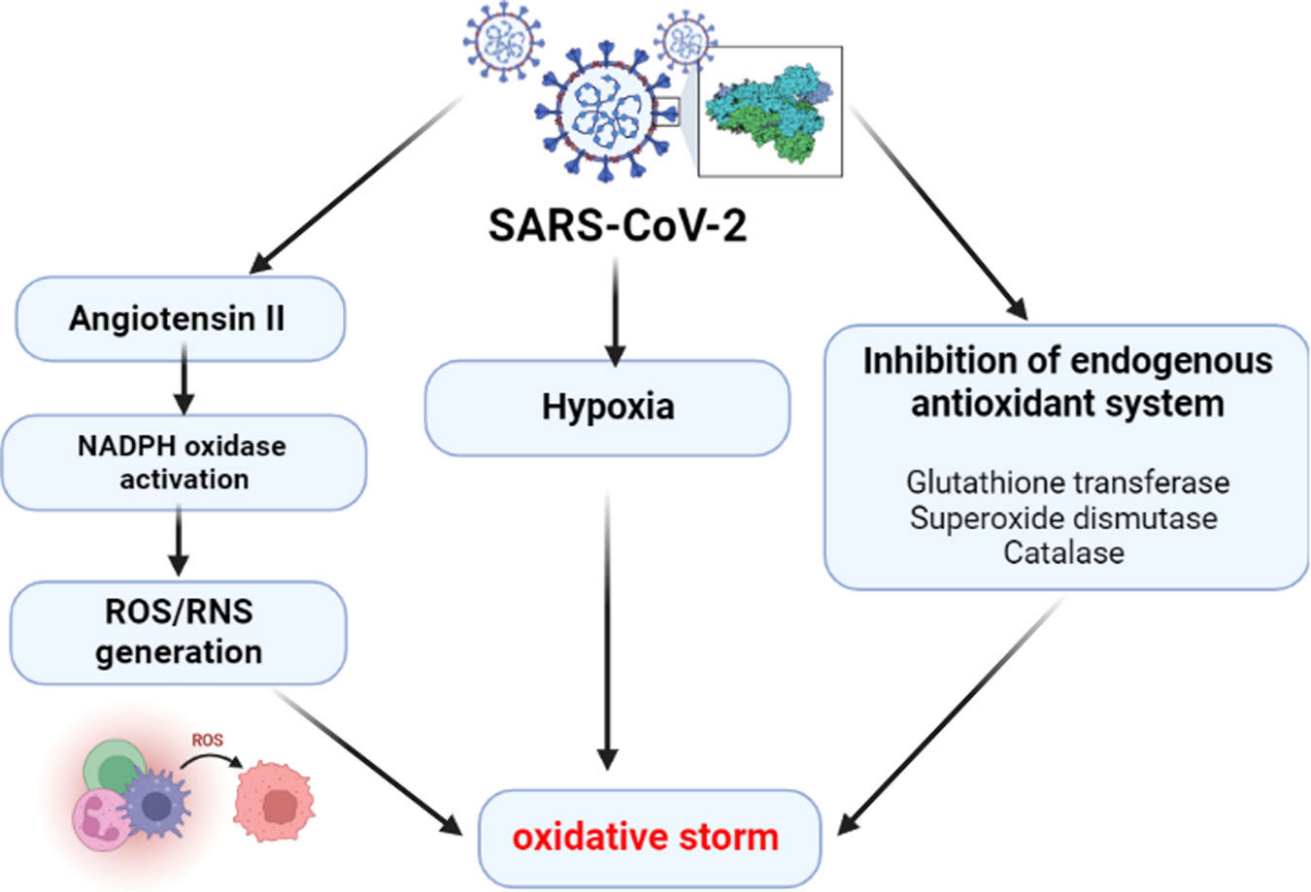
Oxidative stress in Covid‐19: SARS‐CoV‐2 infection lead to hypoxia, dysregulation of renin‐angiotensin system (RAS), overproduction of reactive oxygen species (ROS)/reactive nitrogen species (RNS), and inhibition of endogenous antioxidant system with subsequent oxidative storm formation. Covid‐19, coronavirus disease 2019; SARS‐CoV‐2, severe acute respiratory syndrome coronavirus virus type 2.

These verdicts suggest that hyperinflammation, hypoxia, dysregulation of RAS, overproduction of ROS/RNS, inhibition of endogenous antioxidant system and vitamin D deficiency could be the potential mechanism for the development of oxidative storm in Covid‐19 (Figure 4).

## 5 INFLAMMATORY STORM IN COVID‐19

In SARS‐CoV‐2 infection, there are noteworthy changes in the inflammatory cells characterized by neutrophilia, lymphopenia, and esinopenia.^30^, ^140^ A retrospective study involved 45 patients with severe Covid‐19 compared to 12 patients with mild Covid‐19 illustrated that neutrophilia, lymphopenia and esinopenia were associated and correlated with Covid‐19 severity.^140^ Thus, lymphopenia and esinopenia could be predictors of Covid‐19 severity. However, leukopenia and leukocytosis have been reported to be linked with Covid‐19 severity.^13^, ^141^ Excessive infiltration of inflammatory cells into the lung alveolar cells triggers inflammatory changes and the development of ALI/ARDS during SARS‐CoV‐2 infection.^142^ Thus, the migration of inflammatory cells into the lung tissue could explain the development of lymphopenia and esinopenia in Covid‐19.^43^, ^140^, ^143^ Different clinical studies demonstrated that the development of neutrophilia, lymphopenia, esinopenia, and monopenia were correlated with Covid‐19 severity.^144^, ^145^ Higher infiltration of neutrophils triggers the development of ALI/ARDS by releasing neutrophil‐derived molecules which damage lung epithelial and alveolar cells.^31^, ^140^

It has been shown that imbalance and alteration of the neutrophil functions and phenotypes were marked in patients with severe Covid‐19. During the early phase of SARS‐CoV‐2 infection, the neutrophil counts are increased in the nasopharyngeal tissue and later in the distal part of the respiratory epithelium.^44^, ^146^, ^147^ In severe Covid‐19, the immature neutrophil counts together with neutrophil‐derived molecules is increased. Remarkably, neutrophils isolated from Covid‐19 patients had an immunosuppressive effect through inhibition of INF‐γ production and T cell proliferation.^148^ Further, neutrophils in Covid‐19 have the ability to form NETs due to direct stimulation by SARS‐CoV‐2 through neutrophil membrane TLR4. NETs formation is also triggered by a high level of autoantibodies and platelet activation in Covid‐19.^149^, ^150^ The development of NETs increases the risk for the progression of immunothrombosis and pulmonary microthrombosis with the propagation of ALI/ARDS.^150^ Hypoxia in severe ARDS provokes the neutrophils to form NETs with subsequent respiratory failure.^33^, ^151^

Moreover, lymphocyte plays an important role in the regulation of inflammatory response and immune homeostasis. Lymphocytes have antiinflammatory effects against inflammatory disorders in viral infections.^32^, ^146^, ^152^ Lymphopenia in Covid‐19 is related to different mechanisms including direct injury by SARS‐CoV‐2 through expressed ACE2, a decrease in the production of lymphocytes due to the destruction of the lymphoid organ by SARS‐CoV‐2, lymphocyte apoptosis by proinflammatory cytokines, and inhibition of lymphocytes by metabolic disorders induced by SARS‐CoV‐2 infection.^11^, ^118^ Lymphopenia is associated with the propagation of Covid‐19 severity, it is reduced to <20% in severely affected Covid‐19 patients.^152^, ^153^ Therefore, lymphopenia is considered a potential predictor in hospitalized Covid‐19 patients.

Furthermore, the lymphocyte subsets reflect immunoglobulin M (IgM) and immunoglobulin G (IgG) responders and nonresponders after the onset of symptoms. Total T cells, CD4^+^ T cells, and NK cell percentages are linked to the serological response. In addition, our findings suggested that neutrophil absolute counts and neutrophil to lymphocyte ratio may be precious predictors of IgM or IgG antibody response.^154^ Of interest, IgG and IgM responses in Covid‐19 patients are very poor. As a result, the investigation of different lymphocyte subsets that could trigger significant antibody responses in Covid‐19 patients is crucial.^154^ Furthermore, the level of neutrophil‐to‐lymphocyte ratio was found to be significantly lower in the IgM antibody and IgG antibody responder groups compared to the IgM antibody and IgG antibody nonresponder groups.^144^ In addition, the humoral immune response may be correlated with protection; the evaluation of neutralizing antibodies is more important.^144^ Ever since the neutralizing activities of the detected IgG antibodies remained unknown, though detected an antibody against the N protein of SARS‐CoV‐2; therefore, more studies should be conducted on the detection of antibodies against the S protein along with the detailed analysis of immune cell compositions, to evaluate patient's recovery stage comprehensively.

Of note, eosinophils improve host defense mechanisms against different viral infections including influenza and RSV.^155^, ^156^, ^157^, ^158^ As well, eosinophils have antiviral activity against the parainfluenza virus.^156^, ^159^ In severe SARS‐CoV‐2 infection, eosinopenia is developed in parallel with the reduction of lymphocytes and platelets.^160^, ^161^ For example in a cohort study, eosinophil counts were reduced at the time of admission from patients with severe Covid‐19 that ultimately return to normal at the time of recovery. However, eosinophil counts remain low in Covid‐19 patients with fatal complications.^162^ Thus, eosiopenia in Covid‐19 patients is regarded as an indicator for the development of disease severity and complications.

Moreover, decreasing basophil counts could be a potential prognostic tool in the assessment of Covid‐19 severity. Basopenia in severe SARS‐CoV‐2 infection indicates hyperacute response in severe airway inflammation.^163^ Basophil promotes an immune response against SARS‐CoV‐2 infection. The development of basopenia in severe SARS‐CoV‐2 infection might be by a similar mechanism to lymphopenia. Basopenia impairs immunes' responses and antibody production during SARS‐CoV‐2 infection and is correlated with Covid‐19 severity.^52^, ^163^ A study from Wuhan, China, included 128 Covid‐19 patients and showed that the development of basopenia was present in 13.39% in the first 3 days of the recruited patients.^164^

Notably, the monocytes contribute to the regulation of systemic adaptive and innate immune responses during SARS‐CoV‐2 infection. Herein, circulating monocyte count reflects the state of immune response and systemic hyperinflammation.^165^ Increasing macrophage activation in Covid‐19 augments the migration of circulating monocytes into the inflamed tissues with the development of monocytopenia in severely affected patients.^21^, ^165^, ^166^

**Figure 5:**
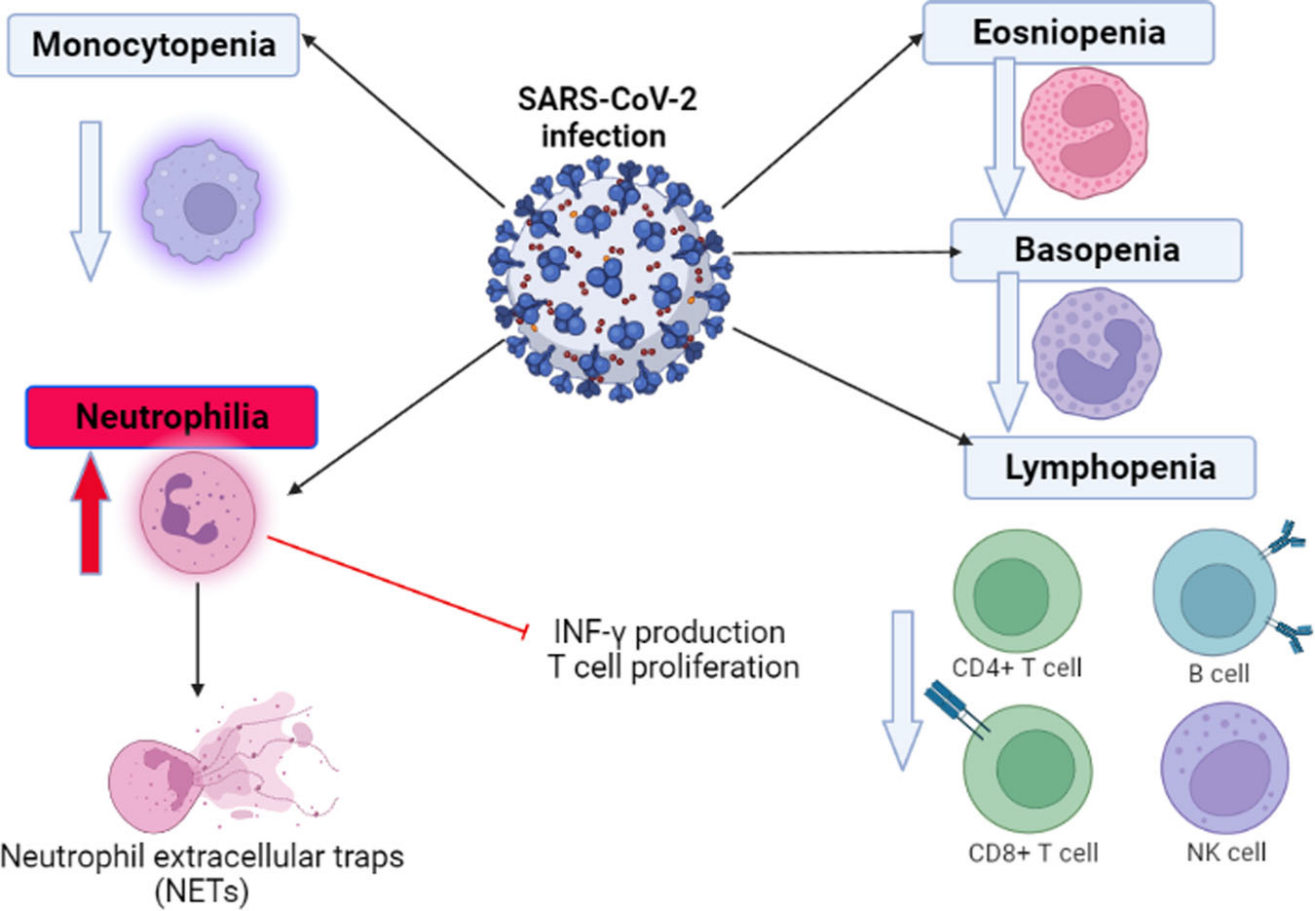
Inflammatory cells in Covid‐19: SARS‐CoV‐2 triggers activation of neutrophils and other blood inflammatory cells leading to eosnipenia, basopenia, lymphopenia, and monocytopenia with neutrophilia and development of neutrophil extracellular traps (NETs). NETs inhibit interferon‐gamma (INF‐γ) production with attenuation of T cell proliferation. Covid‐19, coronavirus disease 2019; SARS‐CoV‐2, severe acute respiratory syndrome coronavirus virus type 2.

Taken together, the inflammatory storm is developing in Covid‐19 due to the development of neutrophilia, lymphopenia, eosinopenia, and monocytopenia with subsequent dysregulation of inflammatory immune response (Figure 5).

## 6 TS IN COVID‐19

Kitchens in 1998 was the first author to use the TS term in his case‐reported study.^167^ Patients with a single thrombotic event may develop subsequent frequent distal thromboembolic complications. The primary thrombotic event may initiate the activation of multiple cascade pathways which induce progressive thrombosis termed TS.^167^ TS represents a serious prothrombotic phenotype with multiple thrombotic complications affecting many vascular beds over a short period.^168^ TS is common in patients with antiphospholipid syndrome, heparin‐induced thrombocytopenia, myeloproliferative disorders, malignancy and sepsis.^168^ TS is mainly treated by aggressive anticoagulants, though TS may resume following the interruption of anticoagulant treatment.^168^, ^169^

It has been shown that thrombotic complications are common in Covid‐19 and correlated with disease severity.^169^ Thrombotic complications and hypercoagulation in Covid‐19 are believed to develop in response to SARS‐CoV‐2 infection‐induced hyperinflammation which causes arterial and venous thromboembolic disorders like limb ischemia and myocardial infarction.^169^ Hence, thrombotic complications in Covid‐19 are regarded as an emergency condition that needs prompt management to prevent the development of fatal outcomes.

The potential mechanism of hypercoagulation and thrombosis in Covid‐19 is related to different mechanisms. Exaggeration of AngII and proinflammatory cytokines in severe SARS‐CoV‐2 infection can induce endothelial dysfunction and progression of thrombotic events.^169^, ^170^ Endothelial injury by SARS‐CoV‐2 infection together with platelet activation and leukocyte activation provokes the release of tissue factors with subsequent initiation of thrombotic cascades.^171^ SARS‐CoV‐2 infection together with high circulating tissue factor stimulates activation of clotting factor VII.^172^ Kamel et al.^173^ illustrated that hyperthrombotic milieu is common in severely affected Covid‐19 patients. Endothelial injury is considered the mainstay and cornerstone in the extension of micro/macrothrombosis in Covid‐19.^173^ Endothelial injury reduces the expression of endothelial anticoagulant proteins like von‐Weilbrand factor, thrombomodulin and glycocalyx.^173^, ^174^, ^175^ The markers of endothelial injury are increased in severely affected patients.^173^, ^176^ In addition, impairment of the endogenous anticoagulant system and inhibition of the fibrinolytic system have been demonstrated in patients with severe Covid‐19.^176^ Furthermore, the progression of thrombotic microangiopathy may further augment thromboembolic complications.^177^

Moreover, complement activation and development of immunothrombosis could be the possible mechanism for coagulopathy in Covid‐19. Complement cascades promote the activation of clotting factors with the induction of endothelial injury.^178^ Likewise, the antiphospholipid syndrome may develop in highly susceptible Covid‐19 induces progression of thrombotic complications.^179^ Different clinical studies reported that the most severely affected Covid‐19 patients had a high level of D‐dimer with thrombotic complications, and presented with ischemic stroke, pulmonary embolism, and cardioembolic events.^180^, ^181^

**Figure 6:**
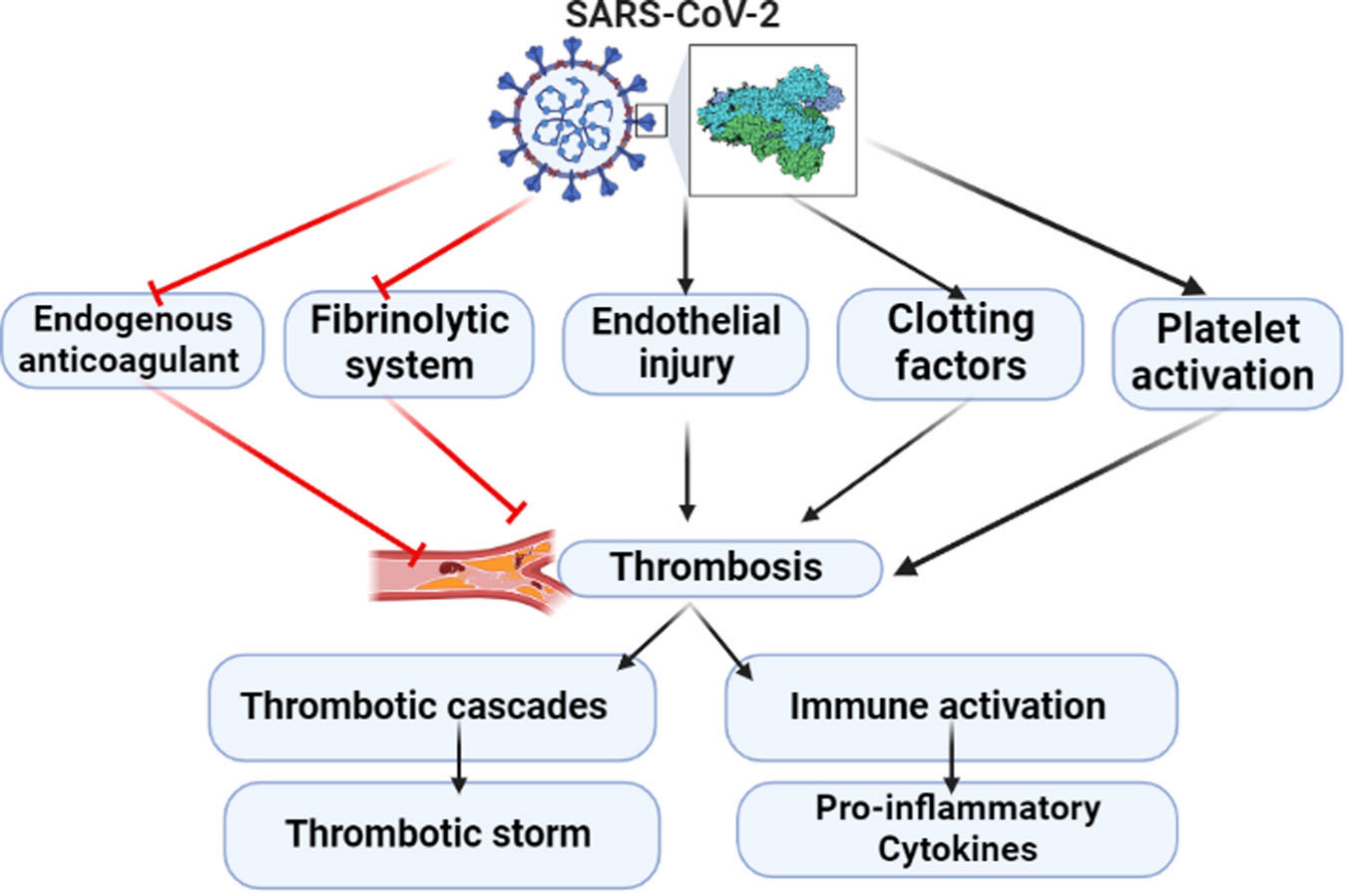
Thrombotic storm in Covid‐19: SARS‐CoV‐2 infection triggers activation of the coagulation system and platelet activation with inhibition of fibrinolytic and endogenous anticoagulant systems. These changes provoke the development of thrombosis which stimulate thrombotic cascades and the release of proinflammatory cytokines leading to the propagation of a thrombotic storm. Covid‐19, coronavirus disease 2019; SARS‐CoV‐2, severe acute respiratory syndrome coronavirus virus type 2.

In this condition, dysregulation of clotting, anticoagulant, and fibrinolytic systems vis‐a‐vis complement activation and development of antiphospholipid syndrome could be the possible pathways for the progression of TS in Covid‐19 (Figure 6).

## 7 MIXED STORM (MS) IN COVID‐19

A MS represents the final storm from the interrelated CS, TS, oxidative storm, lipid storm, and inflammatory storm leading to ALI/ARDS and systemic complications. In fact, CS which has a wide range of propaganda in Covid‐19 did not develop alone, but its progress beside the progression of other types of storms. This new term was not previously mentioned in the context of Covid‐19, though it was suggested by us to give a clearer picture of the interacted storms.

CS with high levels of proinflammatory cytokines can induce endothelial injury with the propagation of thrombotic events and the development of ALI/ARDS.^182^ As well, proinflammatory cytokines mainly TNF‐α and IL‐6 promote coagulation and thrombotic events which also trigger the activation and release of proinflammatory cytokines.^182^ Jose et al.^183^ found that thrombin exerts an inflammatory response through the activation of proteinase‐activated receptors which also inhibit endogenous anticoagulant and fibrinolytic systems. Thus, targeting proteinase‐activated receptor antagonists may block the bridge between coagulation and inflammation in Covid‐19^182^ with suppression of the development of CS and TS. Clotting factors which are stimulated by the proinflammatory cytokines, also act as chemoattractants of immune cells to release more proinflammatory cytokines.^184^ IL‐1 expressed by activated platelets stimulates the release of proinflammatory cytokines.^184^ These findings might explain the close interaction and relationship between CS, inflammatory storm, and TS in Covid‐19.

Furthermore, the development of hypoxia in severe Covid‐19 can induce the generation of ROS which activate the release of proinflammatory cytokines which in turn stimulates the macrophages to produce more ROS in a vicious cycle.^185^ This interaction leads to the development of CS and oxidative storm with the progression of MOI.^185^ Likewise, a higher concentration of ROS activates NF‐κB and NLRP3 inflammasome with induction release of proinflammatory cytokines.^150^ Oxidative storm with highly generated ROS and RNS induces activation and recruitment of immune cells with subsequent release of proinflammatory cytokines. As well, ROS and RNS provoke activation of the coagulation system with the propagation of thrombotic events.^186^ Particularly, complement activation, coagulation disorders and activated inflammatory signaling pathway in Covid‐19 increase expression of 5LOX and COX with suppression of anti‐inflammatory resolvin and lipoxins.^100^, ^187^, ^188^ Activation of 5LOX and COX in Covid‐19 increases the synthesis and release of bioactive lipids which augment the recruitment of the inflammatory and immune cells.^66^, ^94^ 5LOX and COX metabolites induce the release of proinflammatory cytokines with activation of plasmin and expression of tissue factors. These changes provoke the development of lipid storm and propagation of pulmonary microthombosis.^189^, ^190^ Noteworthy, activation of 5LOX and COX are linked with the development of oxidative stress.^191^, ^192^ Therefore, stimulation of 5LOX and COX may increase the risk for the development of oxidative storm in Covid‐19.

Furthermore, dysregulation of RAS and exaggerated AngII level in severely affected Covid‐19 patients could be a potential link between different storm types in the development of MS. Remarkably, higher circulating AngII levels are correlated with thrombotic events.^193^ Besides, AngII overexpression triggers oxidative stress and endothelial dysfunction through the release of proinflammatory cytokines with the development of thromboinflammation.^194^ Therefore, AngII could be a potential biomarker of MS.

**Figure 7:**
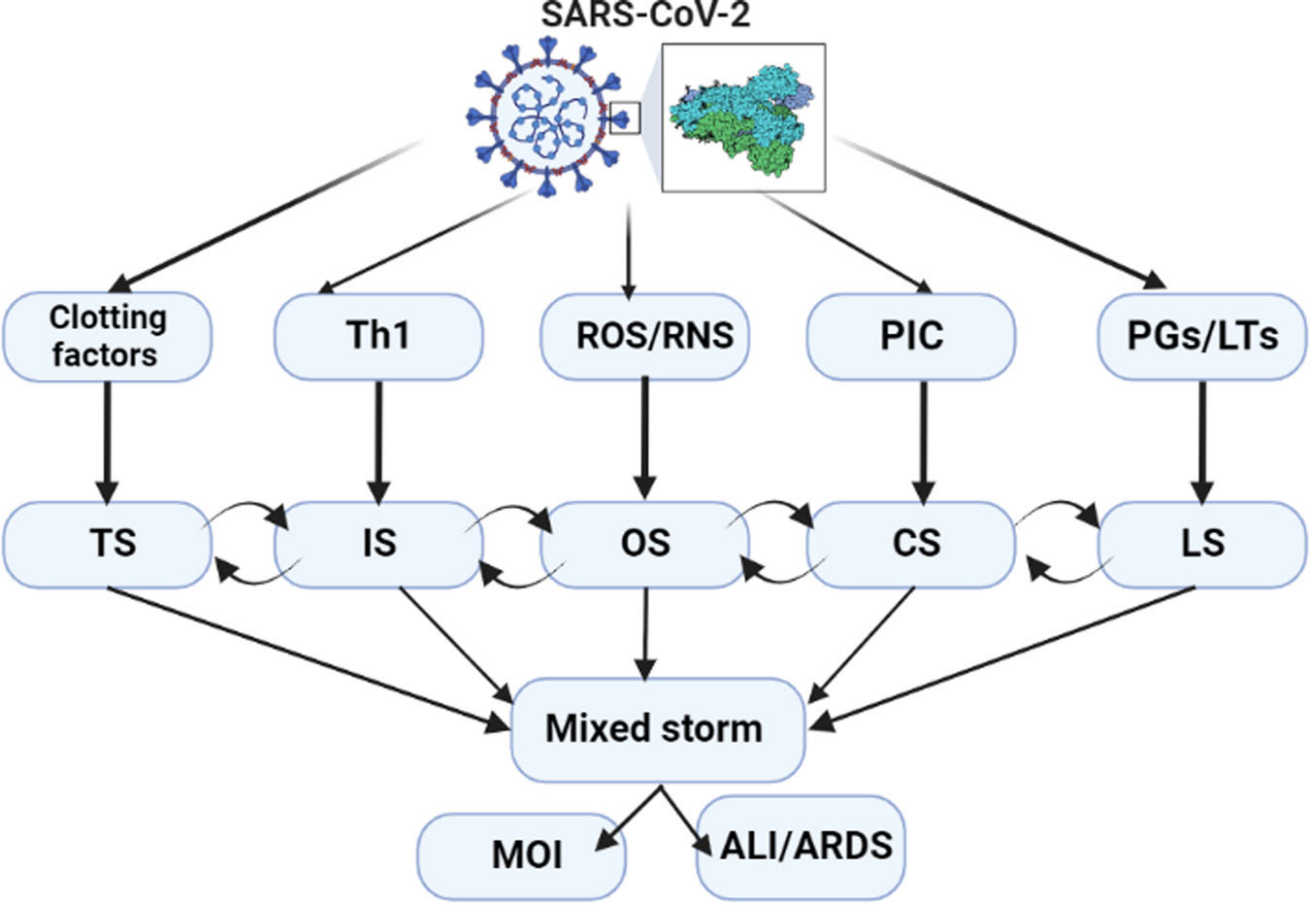
Mixed storm in Covid‐19: SARS‐CoV‐2 activates the release of prostaglandins (PGs) and leukotrienes (LTs) with the development of lipid storm (LS). Stimulation release of proinflammatory cytokines (PIC) and reactive oxygen species (ROS)/reactive nitrogen species (RNS) provoke the development of cytokine storm (CS) and oxidative storm (OS). As well, SARS‐CoV‐2 stimulates T helper cells (Th1) causing inflammatory storm (IS), and activation of clotting factors induces the development of thrombotic storm (TS). The interrelated storms propagate the development of mixed storm with the progression of acute lung injury (ALI), acute respiratory distress syndrome (ARDS) and multiorgan injury (MOI). Covid‐19, coronavirus disease 2019; SARS‐CoV‐2, severe acute respiratory syndrome coronavirus virus type 2.

These observations proposed that MS is propagated in Covid‐19 due to complex interaction between ROS, proinflammatory cytokines, complement activation, coagulation disorders, and activated inflammatory signaling pathway. Therefore, the MS seems to be more appropriate to be related to severe Covid‐19 than CS (Figure 7).

However, specific biomarkers of the MS were not evaluated. Nevertheless, this review suggested a new term in the pathogenesis of SARS‐CoV‐2 infection that may be criticized, so we welcome any commentary regarding this review and a new term.

Taken together, specific storm types and their mediators are intricate in the development and progression of tissue injury, though the interaction between different storm types triggers the development of MS (Table 2).

**Table 2:** Storm types and their mediators.

Type of storm	Mediators
Cytokine storm (CS)	Interleukin (IL)‐1β, IL‐6, IL‐8, **m**onocyte Chemoattractant Protein‐1 (MCP‐1), **n**uclear factor kappa B (NF‐κB) and NLR Family Pyrin Domain Containing 3 (NLRP3) inflammasome.
Lipid storm (LS)	Thromboxan A2 (TXA2), Prostaglandin E2 (PGE2), oxylipins, LeukotrieneB4 (LTB4), LTE4, low‐density lipoprotein (LDL‐c), high‐density lipoprotein (HDL‐C), ApoA, sphingolipids and glycerophospholipids.
Oxidative storm (OS)	Malondialdehyde, reactive oxygen species, **n**icotinamide adenine dinucleotide phosphate (NADPH) oxidase and Vitamin D
Inflammatory storm (IS)	Lymphocytes and ILs.
Thrombotic storm (TS)	D‐dimer
Mixed storm (MS)	Unidentified

## 8 CONCLUSION

Covid‐19 may progress with systemic complications due to direct SARS‐CoV‐2 cytopathic effects and related hyperinflammation and hypercytokinemia with the development of CS. SARS‐CoV‐2 infection prompts various storm types including CS, inflammatory storm, lipid storm, TS and oxidative storm. These storms are not developing alone since there is a close relationship between them. Consequently, the MS seems to be more appropriate to be related to severe Covid‐19 than CS, as it develops in Covid‐19 due to the intricate interface between ROS, proinflammatory cytokines, complement activation, coagulation disorders, and activated inflammatory signaling pathways. Furthermore, inflammatory and oxidative storms increase the risk for the development of CS. Diverse biomarkers reflect the severity of CS, though IL‐6 is the main biomarker intricate in the progression of CS. Also, oxylipins which reflect the development of lipid storm are increased in severely affected Covid‐19 patients due to hyperinflammation and oxidative stress. Thus, proinflammatory oxylipins are augmented in Covid‐19 patients due to defects in the metabolic pathway for the production of antiinflammatory and proresolving oxylipins. Other biomarkers like D‐dimer and MDA in thrombotic and oxidative storms respectively. Particularly, most of these biomarkers are increased concurrently in severely affected Covid‐19 patients suggesting the development of a MS. This review cannot give the ultimate conclusion for the mechanistic role of different storm types in the development of MS. In this regard, experimental, preclinical, and clinical studies are needed to approve the pathogenic profile and related biomarkers of the MS in Covid‐19.

## AUTHOR CONTRIBUTIONS

**Hayder M. Al‐Kuraishy**, **Ali I. Al‐Gareeb** and **Ali K. Al‐Buhadily**: conceptualization and writing of original draft. **Athanasios Alexiou**, **Marios Papadakis**, **Hebatallah M. Saad**, and **Gaber El‐Saber Batiha**: preparation of figures; writing, correcting, and amending of the article. **Basil Mohammed Alomair** and **Majed Ayed Alshammari**: editing and polishing of the manuscript and responding to reviewers' comments. All authors contributed significantly to the manuscript and approved the submitted version.

## CONFLICT OF INTEREST STATEMENT

The authors declare no conflicts of interest.

## Data Availability

Not applicable.
